# Long-term efficacy of traditional Chinese medicine combined with chemotherapy for advanced non-small cell lung cancer: a systematic review and meta-analysis of reconstructed individual patient data over 3 years

**DOI:** 10.3389/fphar.2026.1818515

**Published:** 2026-07-02

**Authors:** Yixuan Guo, Dan Wang, Xuexiu Jia

**Affiliations:** 1 College of Acupuncture-Moxibustion and Tuina, Shandong University of Traditional Chinese Medicine, Jinan, China; 2 College of Traditional Chinese Medicine, Shandong University of Traditional Chinese Medicine, Jinan, China; 3 Affiliated Hospital of Shandong University of Traditional Chinese Medicine, Department of Pediatric Tuina, Jinan, China

**Keywords:** advanced non-small cell lung cancer, reconstructed individual patient data, restricted mean survival time, time-varying hazard ratio, traditional Chinese medicine

## Abstract

**Introduction:**

To evaluate the 3-year efficacy of combining traditional Chinese medicine with chemotherapy in the treatment of advanced non-small cell lung cancer, taking into account time-dependent survival effects.

**Methods:**

A systematic review and meta-analysis was conducted following PRISMA guidelines, including randomized controlled trials and observational studies that compared chemotherapy plus traditional Chinese medicine with chemotherapy alone. Individual patient data were reconstructed from Kaplan–Meier curves. For the primary endpoints of overall survival and progression-free survival, one-stage analyses employed frailty Cox models when the proportional hazards assumption held; otherwise, Royston-Parmar models incorporating time-varying effects were used. Restricted mean survival time over 0–36 months was estimated, and a two-stage random-effects model was applied for sensitivity analysis.

**Results:**

Eighteen studies (12 RCTs and 6 cohort studies) were included, with individual patient data reconstructed from 15 overall survival and 7 progression-free survival curves. The proportional hazards assumption was violated for overall survival; time-varying modeling revealed significantly better overall survival with chemotherapy plus traditional Chinese medicine therapy, with a 3-year restricted mean survival time difference of 4.149 months. In the personalized treatment subgroup, CT + TCM showed a pronounced early survival benefit, a similar benefit was observed in the fixed-regimen subgroup. For progression-free survival, combination therapy significantly improved it (HR = 0.85, 95% CI 0.77–0.93, P < 0.001), corresponding to a restricted mean survival time difference of 1.582 months. In the fixed-regimen subgroup, CT + TCM significantly improved PFS (HR = 0.85, 95% CI 0.77–0.94, P < 0.05), while in the personalized TCM subgroup the PFS benefit was not statistically significant (HR = 0.85, 95% CI 0.67–1.07, P = 0.17).

**Conclusion:**

The addition of traditional Chinese medicine to chemotherapy is associated with improved overall and progression-free survival in advanced non-small cell lung cancer, although the overall survival benefit diminishes over time.

**Systematic Review Registration:**

https://www.crd.york.ac.uk/prospero/ Identifier CRD420261280359.

## 1 Introduction

Advanced non-small cell lung cancer (NSCLC) remains a leading cause of cancer mortality worldwide (Miller et al., 2020; Sung et al., 2021). Over 75% of patients are diagnosed at advanced stages with poor prognosis, and the 5-year overall survival rate is only about 5.2% (Goldstraw et al., 2016). Although systemic chemotherapy (CT) continues to play a central role in many settings (Riely et al., 2024), long-term outcomes remain limited, and treatment-related symptom burden and functional decline frequently complicate clinical management (Kolek, 2014). Consequently, integrative approaches are increasingly used alongside conventional therapy, particularly in East Asia (Jin et al., 2021).

Traditional Chinese medicine (TCM), especially Chinese herbal medicine (CHM), is commonly combined with chemotherapy in practice, aiming to improve tumor control, alleviate adverse effects such as bone marrow suppression and gastrointestinal disturbances, and support patients’ overall condition (Xi et al., 2025). Recent meta-analyses have demonstrated that these combinations may enhance clinical efficacy (McCulloch et al., 2006; Wen et al., 2020; Qiao et al., 2023), and real-world evidence from large cohorts with up to 10-year follow-up has also suggested potential long-term survival advantages (Qin et al., 2025). However, most existing studies have relatively short follow-up periods, limiting their ability to assess long-term efficacy. Second, survival effects may be non-constant over time; the impact of TCM could vary across different phases of treatment and disease progression. Third, individual studies differ substantially in TCM formulations, chemotherapy regimens, and patient populations, making pooled estimates difficult to interpret without reconstructing individual-level data (Guyot et al., 2012).

Therefore, we performed a systematic review and meta-analysis based on 3-year reconstructed individual patient data (IPD) from Kaplan–Meier curves to evaluate the long-term efficacy of chemotherapy plus TCM versus chemotherapy alone in advanced NSCLC. By reconstructing time-to-event data and applying flexible parametric models that accommodate time-varying effects, we aimed to provide more nuanced estimates of survival benefits over a 3-year horizon and to clarify the temporal pattern of treatment effects.

## 2 Methods

### 2.1 Literature search and study selection

This study is a systematic review and meta-analysis based on the reconstruction of individual patient data from Kaplan-Meier (K-M) curves. It follows the Preferred Reporting Items for Systematic Reviews and Meta-Analyses (PRISMA) guidelines (Page et al., 2021) and was prospectively registered with PROSPERO (registration number: CRD420261280359).

A systematic search was conducted in both Chinese and English databases, including PubMed, EMBASE, the Cochrane Library, Web of Science (WOS), as well as the China National Knowledge Infrastructure (CNKI), Wanfang, and Weipu (VIP) databases. The search period covered the period from the inception of each database to February 2026. The search strategy combined MeSH and free-text terms related to NSCLC, chemotherapy, and TCM. The strategy was adapted as necessary to fit the specific features of each database.

The inclusion criteria were predefined in accordance with the PICOS (Population, Intervention, Comparison, Outcomes, Study design) framework (Amir-Behghadami and Janati, 2020):

Population (P): Adult patients with pathologically confirmed advanced NSCLC.

Intervention (I): The experimental group received standard systemic chemotherapy combined with TCM (administered concurrently with chemotherapy).

Comparison (C): The control group received the same chemotherapy regimen.

Outcomes (O): Studies were required to report follow-up data on overall survival (OS) and progression-free survival (PFS), and to provide clear and complete K-M survival curves for at least one of these endpoints, enabling IPD reconstruction.

Study design (S): Randomized controlled trials (RCTs) and observational studies.

Two researchers independently performed the literature screening. Initially, studies were screened based on titles and abstracts, followed by a full-text review. Disagreements were resolved through discussion, and when necessary, a third researcher was consulted to reach a consensus.

### 2.2 Data extraction and risk of bias assessment

Two reviewers independently extracted the following data: basic study characteristics (author, year, journal, center, country, study design, study period, total sample size, number of patients in the CT group, number of patients in the CT + TCM group, and treatment regimen), baseline patient information (mean age, proportion of male patients, proportion of patients with stage IV disease, baseline performance status, proportion of patients with adenocarcinoma), and key data required for survival analysis (K-M curve images, number-at-risk tables, and event information) (Liu et al., 2021). Following data extraction, cross-checking was performed to ensure consistency and completeness.

The risk of bias in RCTs was assessed using the Cochrane Risk of Bias 2 (RoB 2) tool (Sterne et al., 2019), while observational studies were evaluated using the Risk Of Bias In Non-randomized Studies - of Interventions (ROBINS-I) tool (Sterne et al., 2016). All assessments were conducted independently by two reviewers, and disagreements were resolved through discussion.

### 2.3 Individual patient data reconstruction

We reconstructed individual patient data, including survival time and censoring status for each patient, from published K-M curves using the ‘IPDfromKM’ package in R (Liu et al., 2021). This approach integrates coordinate data extracted from the survival curves with the number at risk tables to reverse-engineer the original survival data, thereby generating an IPD dataset suitable for individual-level survival analysis.

### 2.4 One-stage analysis

The reconstructed IPD from each original study were aggregated to construct a unified dataset for analysis. The primary outcomes were overall survival and progression-free survival. Statistical analyses were performed using R (version 4.2.1), primarily employing the ‘survival’ (3.5.0), ‘survminer’ (0.4.9), and ‘rstpm2’ (1.5.2) packages (Royston and Parmar, 2002; Therneau, 2026; Kassambara et al., 2021).

First, Kaplan-Meier curves with 95% confidence intervals (CIs) were estimated and plotted for both groups based on the pooled data, with the number at risk presented at 0, 6, 12, 18, 24, 30, and 36 months. Visual inspection of these curves was conducted to preliminarily assess the plausibility of the proportional hazards (PH) assumption. To control for between-study heterogeneity,a Cox proportional hazards model with a shared frailty term at the study level was employed to estimate the overall treatment effect (Gutierrez, 2002). In addition, a stratified Cox proportional hazards model, with study as the stratification factor (Gray, 2002), was also fitted, and the results were retained for sensitivity analysis. Formal testing of the PH assumption was performed using the Grambsch-Therneau method for both global and variable-specific tests (Grambsch and Therneau, 1994), complemented by visual diagnostic plots of Schoenfeld residuals.

If the PH assumption was not violated (global test P > 0.05 and no systematic trend in the residual plots) (Ananthakrishnan et al., 2021), hazard ratios (HRs) with 95% CIs from the frailty Cox model were reported. If the PH assumption was violated (Verbeeck and Saad, 2024), a flexible parametric survival model (Royston-Parmar model) (Royston and Parmar, 2002) was fitted using the ‘stpm2’ function from the ‘rstpm2’ package. This model characterized the baseline hazard function using restricted cubic splines on the log cumulative hazard scale and incorporated a time-varying coefficient (tvc) for the treatment variable to allow the treatment effect to change over time. Based on the fitted model, survival probability curves with 95% CIs for both the CT and CT + TCM groups over 0–36 months were predicted. The time-varying hazard ratio (HR(t)) was estimated, and the HR(t) curve with 95% CI over 0–36 months was plotted, with (HR = 1) as the reference line. Point estimates and 95% CIs for HR(t) were reported at 0, 6, 12, 18, 24, 30, and 36 months.

As a confirmatory analysis, the difference in restricted mean survival time (RMST) over 0–36 months, defined as ΔRMST = RMST_CT+TCM_−RMST_CT_, was calculated, with its 95% CI estimated using the delta method (Royston and Parmar, 2011). This provided supplementary and more robust evidence regarding the treatment effect. Additionally, subgroup analyses were performed using the one-stage approach within studies categorized by whether personalized traditional Chinese medicine treatment was administered, to explore treatment effects within these specific subgroups.

### 2.5 Two-stage analysis

As a sensitivity analysis, the hazard ratios and their standard errors for each original study were re-estimated based on the reconstructed IPD. A two-stage random-effects model was then used to pool the overall effect sizes (Tierney et al., 2007). Studies were categorized into fixed-regimen and individualized treatment subgroups based on whether personalized traditional Chinese medicine treatment was administered. Within each subgroup, effect sizes were pooled using the restricted maximum likelihood (REML) method, and pooled HRs with 95% CIs and the I^2^statistic were calculated. A forest plot incorporating subgroup information was generated to display individual study effects, within-subgroup pooled effects, and the overall pooled effect (Tierney et al., 2007).

Publication bias was assessed using funnel plots and Egger’s test, with a significance threshold of p < 0.10. If funnel plot asymmetry suggested publication bias, further trim-and-fill analysis was to be conducted. Statistical significance was set at a two-sided p < 0.05.

## 3 Results

### 3.1 Study selection and baseline characteristics

**FIGURE 1:**
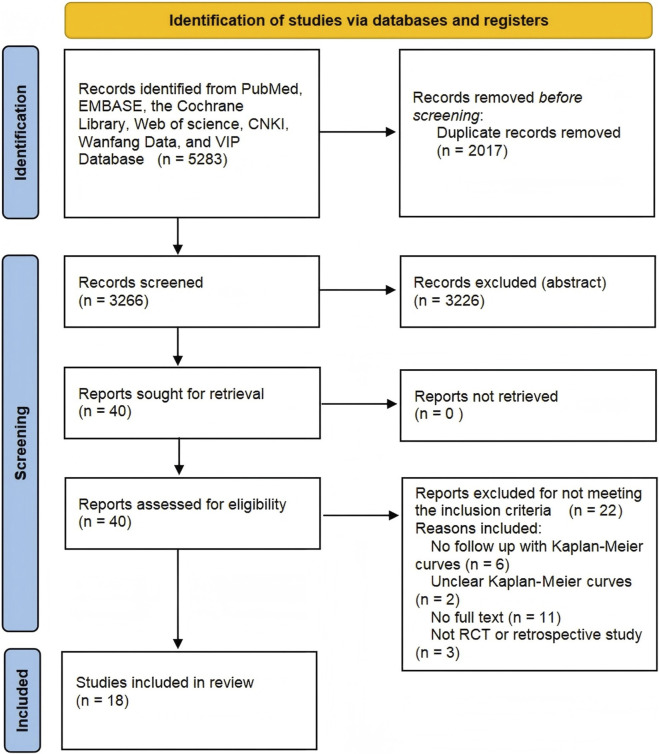
PRISMA flow diagram of study selection.

A total of 5,283 records were identified through database searching (PubMed, EMBASE, the Cochrane Library, Web of Science, CNKI, Wanfang Data, and VIP). After removing 2,017 duplicate records, 3,266 records remained for screening. Title and abstract screening excluded 3,226 records, leaving 40 reports for full-text retrieval; all 40 full texts were successfully retrieved and assessed for eligibility. Ultimately, 18 studies were included in the analysis (Zhou et al., 2005; Chen et al., 2008; Xu et al., 2011; Guo et al., 2012; Xie et al., 2012; Wang et al., 2013; Rong et al., 2014; Li and Li, 2016; Wang et al., 2016; Wang et al., 2018; Wu et al., 2018; Zhang et al., 2018; Guo et al., 2023; Chang et al., 2024), comprising 12 randomized controlled trials (Zhou et al., 2005; Chen et al., 2008; Xu et al., 2011; Guo et al., 2012; Xie et al., 2012; Wang et al., 2013; Rong et al., 2014; Li and Li, 2016; Wang et al., 2016; Wang et al., 2018; Wu et al., 2018; Zhang et al., 2018; Chang et al., 2024) and 6 retrospective cohort studies (Guo et al., 2011; Schad et al., 2018; Huang et al., 2021; Sun et al., 2022; Guo et al., 2023; Chang et al., 2024) (Figure 1). The baseline characteristics of all included studies are summarized in Table 1, and patient characteristics are presented in Table 2 (Supplementary Material).

**TABLE 1:** Characteristics of included studies. CT, chemotherapy; TCM, traditional Chinese medicine.

Author	Year	Journal	Center	Country	Design	Study period	Total sample	CT	CT + TCM	Treatment	Treatment regimen
Zhou et al. (2005)	2005	Chinese Journal of Cancer	Multicenter	China	Randomized Clinical Trial	Dec 2001 - January 2004	195	92	103	He Chan Pian, Shenyi capsules, and herbal decoction	Personalized treatment
Chen et al. (2008)	2008	Chinese Journal of Lung Cancer	Multicenter	China	Randomized Clinical Trial	Oct 2001 - October 2006	100	49	51	Shengmai injection and Gujin granules	Fixed-regimen treatment
Guo et al. (2011)	2011	Integrative Cancer Therapies	Multicenter	China	Retrospective cohort study	Jan 2006 - January 2009	71	30	41	Herbal decoction	Personalized treatment
Xu et al. (2011)	2011	Journal of Cancer Research and Clinical Oncology	Multicenter	China	Randomized Clinical Trial	May 2006 - March 2009	116	53	63	Kangliuzengxiao and Feiyanning decoctions	Fixed-regimen treatment
Guo et al. (2012)	2012	Medical Oncology	Single center	China	Randomized Clinical Trial	May 2008 - March 2010	136	68	68	Astragalus polysaccharide injection	Fixed-regimen treatment
Xie et al. (2012)	2012	Chinese Archives of Traditional Chinese Medicine	Single center	China	Randomized Clinical Trial	May 2009 - May 2011	200	98	102	Herbal decoction	Personalized treatment
Wang et al. (2013)	2013	Chinese Journal of Integrated Traditional and Western Medicine in Critical Care	Single center	China	Randomized Clinical Trial	Jan 2009 - June 2011	120	60	60	Tongbusansheng decoction	Fixed-regimen treatment
Rong et al. (2014)	2014	Chinese Journal of Experimental Traditional Medical Formulae	Single center	China	Randomized Clinical Trial	Jun 2008 - September 2011	197	97	100	Bufei Xiaoji Decoction	Personalized treatment
Li and Li (2016)	2016	Liaoning Journal of Traditional Chinese Medicine	Multicenter	China	Randomized Clinical Trial	Mar 2013 - March 2014	78	39	39	Herbal Decoction	Personalized treatment
Wang et al. (2016)	2016	Modern Oncology	Single center	China	Randomized Clinical Trial	Jan 2008 - October 2012	192	96	96	Shenfu injection	Fixed-regimen treatment
Schad et al. (2018)	2018	PLOS ONE	Multicenter	Germany, Australia	Retrospective cohort study	Feb 2010 - June 2016	158	108	50	Viscum album L	Fixed-regimen treatment
Zhang et al. (2018)	2018	Chinese Journal of Oncology	Multicenter	China	Randomized Clinical Trial	Nov 2013 - November 2016	414	215	199	Shenyi Capsule	Fixed-regimen treatment
Wu et al. (2018)	2018	Chinese Journal of New Drugs	Multicenter	China	Randomized Clinical Trial	Jun 2013 - December 2014	326	170	156	Kang Ai injection	Fixed-regimen treatment
Wang et al. (2018)	2018	Frontiers in Pharmacology	Multicenter	China	Randomized Clinical Trial	Jul 2013 - April 2016	63	30	33	Chinese Herb Medicine Formulas	Fixed-regimen treatment
Huang et al. (2021)	2021	Journal of Interventional Radiology	Single center	China	Retrospective cohort study	Jan 2014 - December 2018	103	71	32	Qingfeixiaoji decoction	Personalized treatment
Sun et al. (2022)	2022	Cancer Research and Clinic	Multicenter	China	Retrospective cohort study	2014–2018	1144	572	572	Xiaoaiping injection	Fixed-regimen treatment
Guo et al. (2011)	2023	Phytomedicine	Multicenter	China	Retrospective cohort study	Jan 2009 - December 2018	318	19	299	Herbal decoction	Personalized treatment
Chang et al. (2024)	2024	Cancer Research and Clinic	Single center	China	Retrospective cohort study	Jan 2016 - December 2018	69	33	36	Kanglixin capsule	Fixed-regimen treatment

**TABLE 2:** Baseline characteristics of the included studies. Values are reported as CT + TCM/CT, NA indicates that the corresponding variable was not reported.

Author	Year	Mean age	Male %	Stage IV %	Baseline KPS	Adenocarcinoma %
Zhou et al. (2005)	2005	57.2/58.0	71.0/80.0	50.0/49.0	71.8/71.6	59.0/64.0
Chen et al. (2008)	2008	60.4/57.8	56.9/67.3	64.7/65.3	72.3/71.3	54.9/46.9
Guo et al. (2011)	2011	NA	NA	NA	NA	NA
Xu et al. (2011)	2011	60.1/62.2	57.1/60.4	76.2/56.6	81.8/82.3	61.9/58.5
Guo et al. (2012)	2012	62.0/62.3	58.8/64.7	60.3/63.2	89.0/88.2	NA
Xie et al. (2012)	2012	NA	68.6/68.4	55.9/57.1	93.6/93.9	54.9/56.1
Wang et al. (2013)	2013	57.2/57.7	70.0/76.7	53.3/48.3	NA	38.3/36.7
Rong et al. (2014)	2014	54.2/52.9	62.0/68.0	NA	73.3/74.4	NA
Li and Li (2016)	2016	62.4/61.7	48.7/53.8	25.6/30.8	NA	46.2/48.7
Wang et al. (2016)	2016	54.7/55.4	65.6/71.9	66.7/72.9	86.0/85.8	50.0/44.8
Schad et al. (2018)	2018	64.5/63.9	55.6/56.0	100.0/100.0	NA	NA
Zhang et al. (2018)	2018	59.0/58.0	66.7/66.5	68.2/70.0	NA	57.1/68.2
Wu et al. (2018)	2018	58.8/58.0	66.7/70.0	84.8/73.3	86.4/86.0	87.9/70.0
Wang et al. (2018)	2018	61.2/60.8	64.3/70.2	50.3/56.7	79.2/80.1	57.3/56.3
Huang et al. (2021)	2021	69.9/69.8	67.6/56.3	NA	NA	NA
Sun et al. (2022)	2022	57.9/57.9	70.1/73.3	95.5/94.1	NA	61.2/60.8
Guo et al. (2023)	2023	NA	NA	100.0/100.0	NA	100.0/100.0
Chang et al. (2024)	2024	55.0/54.5	53.7/58.5	48.8/43.9	NA	46.3/48.8

**FIGURE 2:**
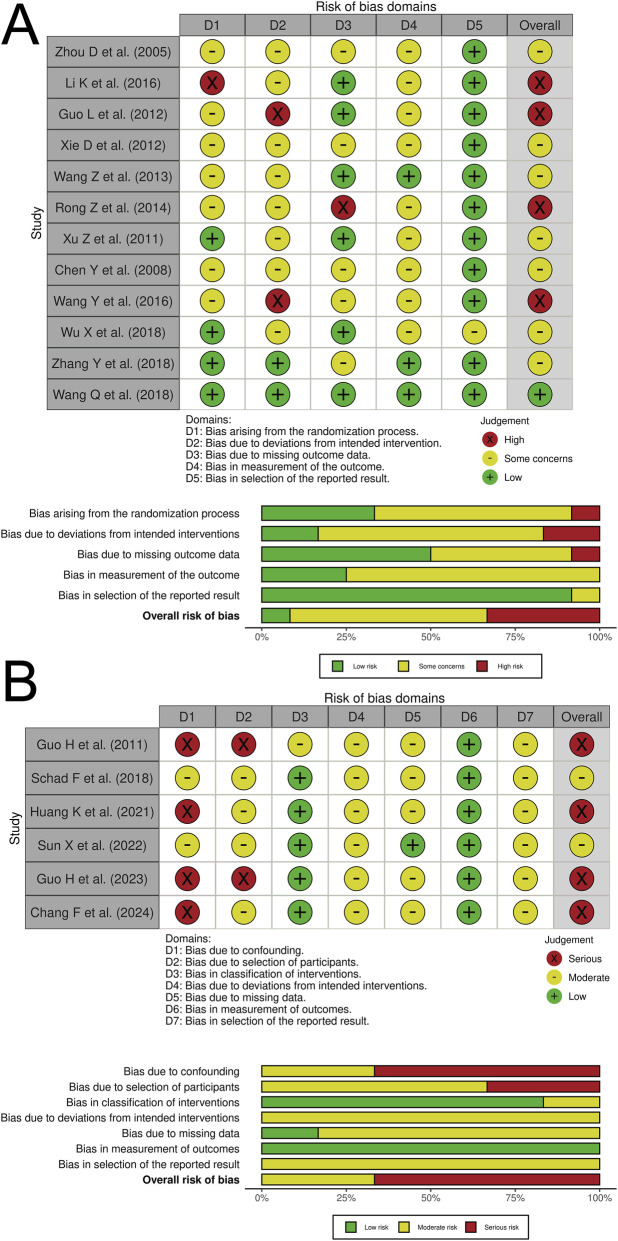
Risk of bias assessment of included studies. **(A)** Risk of bias in randomized trials assessed with RoB 2. Domains: D1, bias arising from the randomization process; D2, bias due to deviations from intended interventions; D3, bias due to missing outcome data; D4, bias in measurement of the outcome; D5, bias in selection of the reported result; Overall, overall risk of bias. Judgments are coded as high risk (red), some concerns (yellow), and low risk (green). Stacked bars summarize the distribution of judgments across domains and overall. **(B)** Risk of bias in non-randomized studies assessed with ROBINS-I. Domains: D1, bias due to confounding; D2, bias in selection of participants; D3, bias in classification of interventions; D4, bias due to deviations from intended interventions; D5, bias due to missing data; D6, bias in measurement of outcomes; D7, bias in selection of the reported result; Overall, overall risk of bias. Judgments are coded as serious (red), moderate (yellow), and low (green). Stacked bars summarize the distribution of judgments across domains and overall.

Risk of bias was assessed using the RoB two tool for randomized trials (Figure 2A) and the ROBINS-I tool for non-randomized studies (Figure 2B). Among the 12 randomized controlled trials assessed by RoB 2, the overall risk of bias was predominantly moderate: 7 studies were judged as having moderate risk, 4 as high risk, and only 1 as low risk. For the 6 observational studies evaluated with ROBINS-I, the overall risk of bias was predominantly high: 4 studies were rated as high risk, 2 as moderate risk, and none as low risk. Overall, the risk of bias in the included evidence primarily stemmed from systematic bias inherent in non-randomized studies and from uncertainties in several domains among the randomized trials.

### 3.2 Individual patient data reconstruction and meta-analysis

Individual patient data were reconstructed from 16 OS curves and 8 PFS curves extracted from the included studies (Supplementary Material).

#### 3.2.1 Overall survival

The reconstructed OS survival curves exhibited notable crossing during follow-up (Supplementary Figure S1A), and the Grambsch–Therneau test (p < 0.05) combined with visualization of Schoenfeld residuals (Supplementary Figures S1B, S1C) indicated violation of the PH assumption. Therefore, a flexible parametric model was employed to predict OS curves for both groups and to estimate time-varying HRs.

**FIGURE 3:**
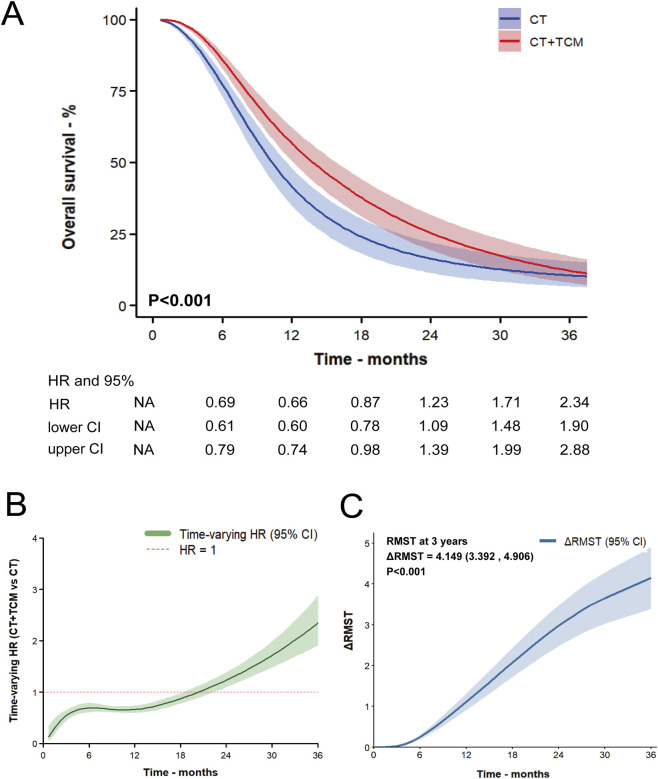
Overall survival comparing chemotherapy (CT) with chemotherapy plus traditional Chinese medicine (CT + TCM). **(A)** Predicted overall survival curves up to 36 months for the CT (blue) and CT + TCM (red) groups; shaded areas represent 95% confidence intervals. Hazard ratios (HRs) at 6, 12, 18, 24, 30, 36 months with 95% CIs are provided beneath the graph. **(B)** Time-varying HR for CT + TCM versus CT with 95% CI. The horizontal dashed line indicates HR = 1. **(C)** Difference in restricted mean survival time (ΔRMST) between the two groups over time with 95% CI. The 3-year ΔRMST estimate is annotated on the graph (ΔRMST = 4.149 months, 95% CI 3.392–4.906, P < 0.001).

Over the 3-year follow-up period, the predicted survival curves demonstrated that CT + TCM was associated with significantly better OS compared with CT alone (P < 0.001). The time-varying analysis confirmed non-proportional hazards: the HR remained below one during early follow-up, favoring CT + TCM, but gradually increased over time and crossed one in later follow-up, indicating that the survival benefit was time-dependent. Consistently, the RMST analysis revealed a significant gain in survival time for the CT + TCM group at 3 years, with a ΔRMST of 4.149 months (ΔRMST = 4.149, 95% CI 3.392–4.906, P < 0.001) (Figures 3A–C).

**FIGURE 4:**
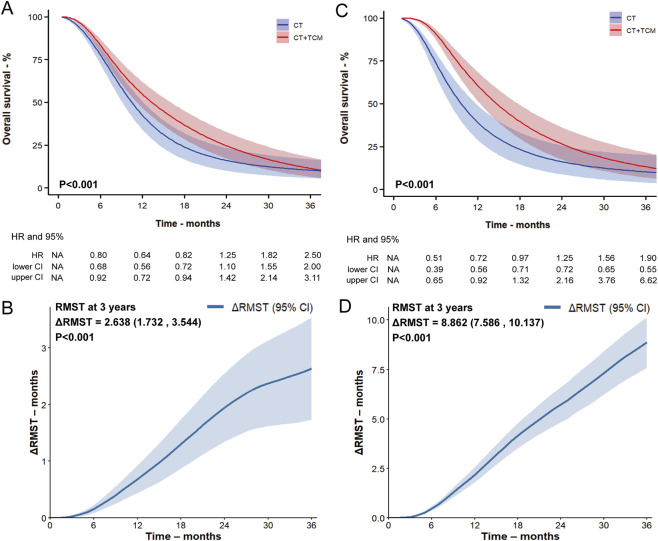
Predicted curves for overall survival (OS) comparing CT + TCM with CT, stratified by treatment regimen. **(A)** Predicted OS curves for the fixed-regimen treatment subgroup. HRs at 6, 12, 18, 24, 30, 36 months with 95% CIs are provided beneath the graph. **(B)** ΔRMST of OS between the CT + TCM and CT groups in the fixed-regimen treatment subgroup over time, shown with the 95% confidence interval (ΔRMST = 2.638, 95% CI: 1.732–3.544, P < 0.001). **(C)** Predicted OS curves for the personalized treatment subgroup. HRs at 6, 12, 18, 24, 30, 36 months with 95% CIs are provided beneath the graph. **(D)** ΔRMST of OS between the CT + TCM and CT groups in the personalized treatment subgroup over time, shown with the 95% confidence interval (ΔRMST = 8.862, 95% CI: 7.586–10.137, P < 0.001).

In the fixed-regimen treatment subgroup (Figure 4A), the CT + TCM group showed a superior survival benefit during early follow-up, although the HRs gradually increased over time. Over the 3-year follow-up period, patients in the CT + TCM group had significantly prolonged RMST compared with the CT group, with an absolute extension of 2.638 months (ΔRMST = 2.638, 95% CI 1.732–3.544, P < 0.001) (Figure 4B). In the personalized treatment subgroup (Figure 4C), a similar survival advantage was observed, characterized by a more pronounced early effect. Specifically, the HR was approximately 0.51 (95% CI 0.39–0.65, P < 0.001) at 6 months and 0.72 (95% CI 0.56–0.92, P < 0.001) at 12 months; thereafter, the HR gradually increased and exceeded 1.0 at later time points. Notably, as illustrated in Figure 4D, the survival benefit in this subgroup was even more striking. Within the 3-year follow-up, patients receiving CT + TCM gained an average of 8.862 additional months of survival compared with those receiving CT alone (ΔRMST = 8.862, 95% CI 7.586–10.137, P < 0.001). Kaplan–Meier curves and residual plots for both groups are presented in Supplementary Figures S2A–S2F. In summary, the subgroup analyses revealed that both fixed-regimen and personalized TCM treatments prolonged overall survival in patients with NSCLC, with the personalized approach demonstrating a superior survival benefit.

**FIGURE 5:**
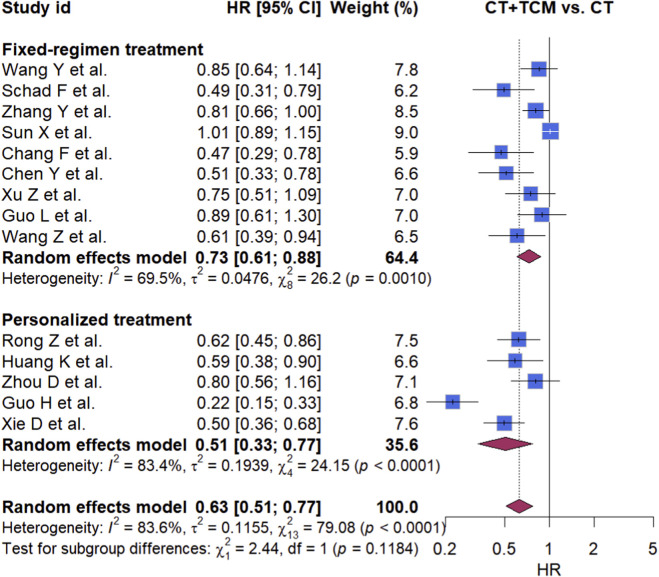
Two-stage sensitivity meta-analysis forest plot for OS (CT + TCM vs. CT), stratified by fixed-regimen and personalized treatment subgroups. The overall estimate shows a significant benefit for CT + TCM with substantial heterogeneity (HR = 0.63, 95% CI 0.51–0.77, I^2^ = 83.6%). Within subgroups, the HR was 0.73 (95% CI 0.61–0.88) for the fixed-regimen group and 0.51 (95% CI 0.33–0.77) for the personalized treatment group. The test for subgroup differences was not significant (p = 0.1184).

A two-stage sensitivity meta-analysis comparing CT + TCM with CT alone, stratified by fixed-regimen versus personalized treatment, showed that CT + TCM significantly improved outcomes overall (HR = 0.63, 95% CI 0.51–0.77), albeit with substantial heterogeneity (I^2^ = 83.6%, p < 0.0001). Subgroup analyses yielded consistent findings: in the fixed-regimen subgroup, CT + TCM remained significantly beneficial (HR = 0.73, 95% CI 0.61–0.88, I^2^ = 69.5%, p = 0.0010); in the personalized treatment subgroup, a significant benefit was also observed (HR = 0.51, 95% CI 0.33–0.77, I^2^ = 83.4%, p < 0.0001). The test for subgroup differences indicated no statistically significant distinction between the two treatment strategies (χ^2^ = 2.44, df = 1, p = 0.1184) (Figure 5). These findings were consistent with the results obtained from the one-stage analysis.

#### 3.2.2 Progression-free survival

**FIGURE 6:**
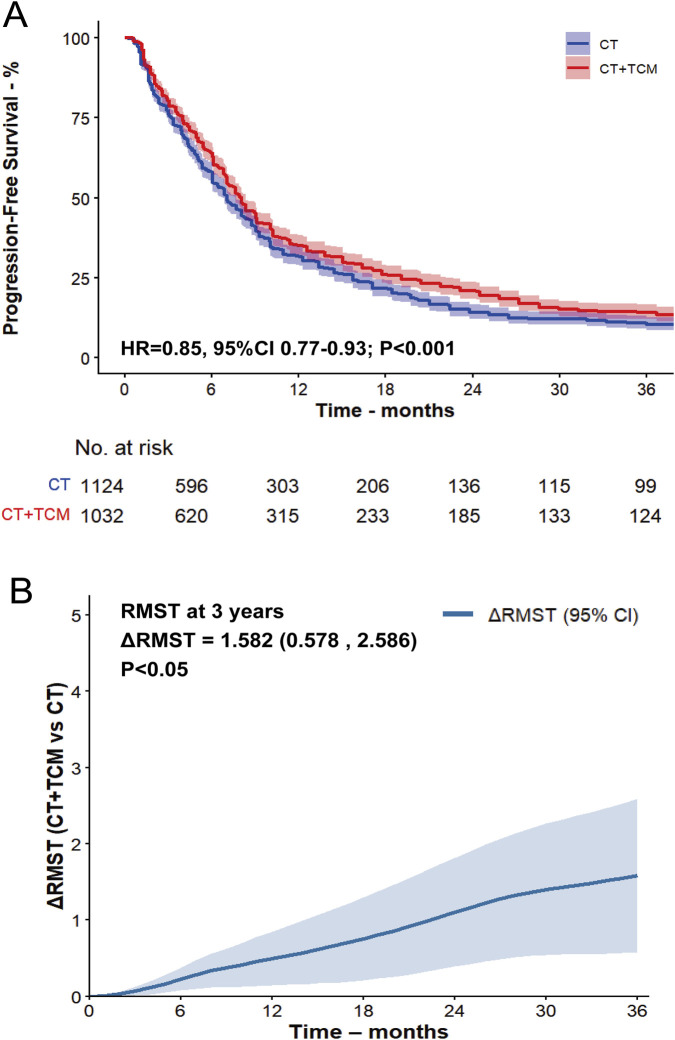
Progression-free survival (PFS) and RMST analysis comparing CT + TCM with CT. **(A)** Kaplan–Meier curves for PFS. Blue and red lines represent the CT and CT + TCM groups, respectively (HR = 0.85, 95% CI 0.77–0.93, P < 0.001). The number of patients at risk at each time point is displayed below the graph. **(B)** ΔRMST (CT + TCM vs. CT) curve over time with 95% CI (ΔRMST = 1.582 months, 95% CI 0.578–2.586, P < 0.05).

Over the 3-year follow-up period, the CT + TCM group demonstrated significantly better PFS compared with the CT group. The Kaplan–Meier curves began to separate early in follow-up, and the proportional hazards assumption was not violated, allowing the use of a frailty Cox model for analysis. The difference was statistically significant (HR = 0.85, 95% CI 0.77–0.93, P < 0.001), indicating that combining TCM with chemotherapy reduced the risk of progression or death by approximately 15%. RMST analysis further supported this finding: at 3 years, the ΔRMST for CT + TCM versus CT alone was 1.582 months (95% CI 0.578–2.586, P < 0.05), suggesting a mean PFS benefit of approximately 1.6 months associated with the combination therapy (Figure 6).

**FIGURE 7:**
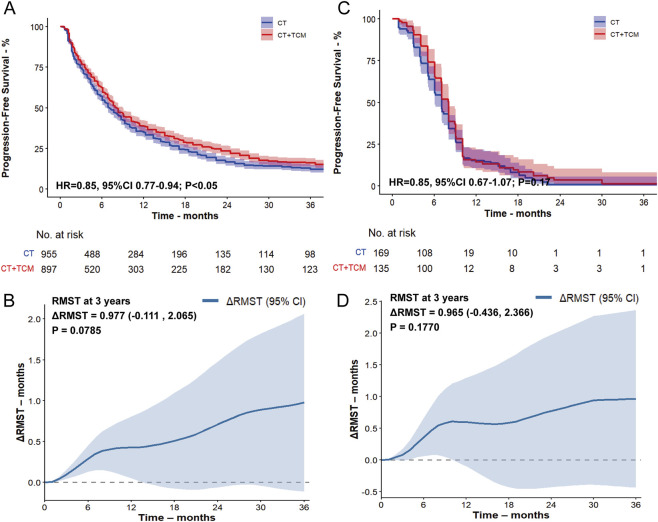
Kaplan–Meier curves for PFS comparing CT + TCM with CT, stratified by treatment regimen. **(A)** PFS curves for the fixed-regimen subgroup (HR = 0.85, 95% CI 0.77–0.94, P < 0.05). **(B)** ΔRMST of PFS between the CT + TCM and CT groups in the fixed-regimen treatment subgroup over time, shown with the 95% confidence interval (ΔRMST = 0.977, 95% CI: 0.111–2.065, P < 0.0785). **(C)** PFS curves for the personalized treatment subgroup (HR = 0.85, 95% CI 0.67–1.07, P = 0.17). **(D)** ΔRMST of PFS between the CT + TCM and CT groups in the personalized treatment subgroup over time, shown with the 95% confidence interval (ΔRMST = 0.965, 95% CI: 0.436–2.366, P = 0.177).

Subgroup analyses further evaluated the PFS benefit associated with different TCM treatment strategies. In the fixed-regimen subgroup (Figure 7A), the PFS curves of the two groups showed a statistically significant separation during the early follow-up period (HR = 0.85, 95% CI 0.77–0.94, P < 0.05), indicating an early progression-free survival advantage for CT + TCM treatment. However, over the entire 3-year observation period, the 95% CI of the difference in RMST crossed zero, and the difference did not reach statistical significance (P > 0.05) (Figure 7B). In the personalized-regimen subgroup (Figure 7C), the PFS curves of the two groups largely overlapped (HR = 0.85, 95% CI 0.67–1.07, P = 0.17); although the RMST in the CT + TCM group was prolonged by 0.965 months compared with the CT group, the difference was also not statistically significant (Figure 7D). Taken together, these findings suggest that although the RMST calculated for the early phase alone may numerically show a trend toward prolonged PFS, this advantage was not sustained over the 3-year overall observation period. Furthermore, the limited sample size of this study may lack sufficient statistical power to definitively confirm this benefit. All residual plots are shown in Supplementary Figures S3A–S3E.

In the subgroup analysis stratified by regimen type using a two-stage random-effects model, CT + TCM was associated with significantly improved PFS compared with CT alone in the overall analysis, with low-to-moderate heterogeneity (HR = 0.83, 95% CI 0.75–0.93, I^2^ = 26.5%, p = 0.226). In the fixed-regimen subgroup, the pooled effect remained significant (HR = 0.82, 95% CI 0.72–0.93, I^2^ = 26.5%, p = 0.2451). In the personalized treatment subgroup, the pooled estimate favored CT + TCM but was not statistically significant, with substantial heterogeneity (HR = 0.82, 95% CI 0.54–1.25, I^2^ = 62.4%, p = 0.1032). There was no evidence of a subgroup difference between the fixed-regimen and personalized approaches (χ^2^ = 0.00, df = 1, p = 0.978). These findings were consistent with the results obtained from the one-stage analysis (Supplementary Figure S4).

#### 3.2.3 Publication bias

Visual inspection of the funnel plot for OS and PFS suggested asymmetry (Supplementary Figures S5A, S5B). This was supported by Egger’s regression test for funnel plot asymmetry (t = −3.83, df = 12, p = 0.0024), indicating evidence of small-study effects. For PFS, formal testing was not conducted because the number of studies was insufficient (n = 7, below the prespecified minimum for Egger’s test), and inference therefore relied on qualitative assessment only. And then, a trim-and-fill adjustment was performed for OS and PFS (Supplementary Figures S5C, S5D).

After adjusting for potential publication bias using the trim-and-fill method, the pooled HR for OS was 0.86 (95% CI 0.67–1.12, p = 0.26, I^2^ = 88.9%), and the pooled HR for PFS was 0.89 (95% CI 0.77–1.03, p = 0.11, I^2^ = 49.4%). Neither endpoint showed statistically significant benefit, and the heterogeneity for OS was extremely high. Therefore, current evidence suggests that publication bias influences the clinical effectiveness of TCM. Any description of a trend toward benefit must clearly state its limitations, avoiding overinterpretation.

## 4 Discussion

This reconstructed individual patient data analysis shows that adding traditional Chinese medicine to chemotherapy improves outcomes in advanced non-small cell lung cancer. Overall survival exhibited a clear time-dependent pattern: the hazard ratio increased over time, with restricted mean survival time analysis yielding an average gain of 4.15 months at 3 years. In frailty Cox models, the hazard of progression or death was reduced by approximately 15% for progression-free survival, corresponding to a 1.6-month restricted mean survival time benefit. Subgroup analyses indicated overall survival advantages for both fixed-formula and personalized strategies, with more pronounced early effects in the individualized therapy group that attenuated over time. Two-stage analyses confirmed the presence of overall survival benefits in both subgroups. For progression-free survival, the benefit was driven primarily by fixed-regimen studies; the personalized subgroup showed a non-significant favorable trend, possibly due to sample size and heterogeneity.

This study observed that the HR for OS crossed one at approximately 20 months, suggesting that the long-term benefit of combining Chinese herbal medicine with chemotherapy may gradually converge with, or even fall below, that of chemotherapy alone in later stages. This phenomenon may be attributed to multiple factors: First, the overall prognosis of advanced NSCLC is extremely poor (Goldstraw et al., 2007). A database–based study indicate that the 3-year OS rate for stage IIIB/IV patients is only 11.7% (Wang et al., 2025), and about 75% of patients have missed the opportunity for curative treatment at diagnosis (Duma et al., 2019). The malignant progression of the disease itself becomes the dominant prognostic factor in later stages. Second, the possibility of potential resistance of tumors to Chinese herbal medicine or its limited efficacy in later treatment phases cannot be ruled out, although high-quality clinical evidence on tumor resistance to Chinese medicine remains scarce. Furthermore, methodological limitations of existing related studies, such as small sample sizes, non-standardized study designs, inconsistent endpoint evaluations, and the lack of uniform standards for composition and quality control of Chinese herbal preparations, may also contribute, to some extent, to the HR reversal and instability of results during later follow-up (Uno et al., 2014). Although the additional effect of Chinese herbal medicine combined with chemotherapy diminishes in the later stages of advanced NSCLC treatment, over the entire 3-year follow-up period, the RMST still captures a clinically meaningful overall survival benefit. This overall benefit may derive from the multifaceted synergistic and supportive roles of Chinese herbal medicine (Ma et al., 2009; Wang et al., 2020). In terms of antitumor mechanisms, conventional chemotherapy acts through DNA damage and cell cycle arrest but is highly prone to inducing multidrug resistance, thereby limiting efficacy (Gu et al., 2025). In contrast, Chinese herbal medicine features multi-component, multi-target characteristics; some of its active ingredients, such as baicalein (Taniguchi et al., 2008), curcumin, and tanshinone (Zhang et al., 2024), have shown potential advantages in reversing chemotherapy resistance by modulating drug transport, restoring apoptosis, and interfering with DNA repair pathways (Maleki Dana et al., 2022). In real-world clinical practice, another core value of Chinese herbal medicine lies in its comprehensive management of chemotherapy-induced toxicities. Chinese herbal medicine is often used to alleviate symptoms such as appetite loss, sleep disturbances, cancer-related fatigue, and gastrointestinal discomfort during chemotherapy (Huang et al., 2025). By effectively reducing chemotherapy-related toxicities (Li J. et al., 2025), Chinese herbal medicine can significantly improve patients’ chemotherapy completion rates and maintain dose intensity (Crawford et al., 2020), thereby enhancing the prognosis of NSCLC patients. This further explains why the combination therapy group achieved superior survival benefits over the entire disease course in this study.

This study observed a time-varying effect in OS that failed to meet the proportional hazards assumption, whereas PFS better satisfied the proportional hazards assumption. This inconsistency between OS and PFS effects is not uncommon (Broglio and Berry, 2009; Hess et al., 2019). Compared with OS, PFS more directly reflects the immediate effect of first-line anti-tumor regimens and is less confounded by subsequent disease progression and post-line treatments (Soria et al., 2010). In this study, the 3-year restricted mean survival time for PFS was approximately 1.6 months, suggesting a modest improvement in disease control overall. The 3-year restricted mean survival time for OS was approximately 4.1 months, potentially indicating that the benefit may derive from better treatment tolerability, fewer severe complications, and a higher probability of receiving and benefiting from subsequent therapies. Mechanistically, Chinese herbal medicine generally does not possess direct, potent cytotoxic effects sufficient to produce a substantial PFS gain. Instead, it transiently alters the trajectory of treatment resistance and disease progression through multi-target, host-mediated effects (Zhou et al., 2023). For example, Astragalus polysaccharides promote dendritic cell maturation and enhance CD8^+^ T cell infiltration into tumors (Liu P. et al., 2025), while compounds from Ganoderma inhibit myeloid-derived suppressor cell expansion and reduce PD-L1 expression in the tumor microenvironment (Bu et al., 2024), thereby delaying the emergence of drug-tolerant persister cells during chemotherapy stress. Meanwhile, classic spleen-strengthening formulas, such as Shenling Baizhu San, have been shown to repair chemotherapy-induced intestinal barrier integrity damage and alleviate systemic cytokine storms (Liu M. et al., 2025). These multiple actions, such as delaying resistance, immune-metabolic regulation, and mitigating chemotherapy toxicity, may yield survival benefits disproportionate to the degree of tumor shrinkage, explaining the discordance between PFS and OS as well as the time-varying pattern of the OS effect. This pattern aligns with the role of Chinese herbal medicine in integrative oncology, where its potential value lies more in supporting systemic status and improving treatment tolerability than merely in direct cytotoxicity s (Mao et al., 2022; Li S. et al., 2025).

The finding that personalized TCM is associated with a more pronounced overall survival benefit warrants careful interpretation, and the following reasons may account for this observation. Personalized treatment emphasizes syndrome differentiation and treatment, allowing adjustment of herbal prescriptions according to the dynamically changing symptom patterns and physiological status during chemotherapy, thereby more effectively alleviating adverse reactions and supporting chemotherapy completion in the early phase (Ji et al., 2025). Studies on TCM syndrome elements have shown that yang deficiency, spleen deficiency, and collapse syndrome are risk elements significantly associated with poor prognosis (Yang et al., 2022), suggesting that early interventions targeting these elements—such as supporting yang, fortifying the spleen—are of considerable value. By the third to fourth chemotherapy cycle, most patients become debilitated with fatigue and delayed immune recovery, such as persistently low CD4^+^ counts and recurrent infections (Cho et al., 2022; Hong et al., 2024). Treatment focus shifts to long-term maintenance, and the benefit of personalized therapy wanes. This attenuation is common to dynamic treatment strategies: like ctDNA-guided adaptive therapy, symptom-based formula adjustment is essentially an adaptive intervention (Lv et al., 2026). Methodological heterogeneity—wide variation in formula composition, dosage, and duration—increases inter-study variability, masks true effects, and accelerates observed benefit attenuation. Personalized regimens often freely combine dozens of herbs at varying doses and durations, hampering standardization and reducing statistical power. Over time, cumulative regimen differences blur the early survival advantage. Thus, the efficacy attenuation of personalized TCM stems from both biological adaptation and inherent methodological limitations.

This study addresses key methodological limitations of previous meta-analyses in integrative oncology. Prior studies have largely relied on study-level summary measures, such as single Cox hazard ratios or event rates at fixed time points, implicitly assuming the proportional hazards assumption holds. Our finding of proportional hazards violation for overall survival suggests that traditional approaches using one hazard ratio for the entire follow-up period may mask clinically meaningful benefits or risks across different follow-up periods. This issue is increasingly recognized in oncology research. By using reconstructed individual patient data, this study enables explicit testing of proportional hazards assumptions and employs Royston-Parmar flexible parametric models with time-varying coefficients to characterize how effects evolve over time. This approach quantifies when benefits appear, how they change, and whether they persist, information often lost in traditional meta-analyses. Furthermore, restricted mean survival time, as an absolute effect measure closer to patient experience, maintains clear clinical interpretability even when the hazard ratio changes direction in later periods.

This study has several important limitations. First, reverse data reconstruction suffers from inherent distortion. The approximate derivation of individual survival data from Kaplan–Meier curves is highly susceptible to limitations in image resolution and missing numbers at risk, leading to magnified measurement errors in the survival trajectory during the long tail of follow-up. Second, Chinese herbal medicine interventions exhibit a “black box” characteristic. Fragmented reporting of core information such as formula composition, dosage, treatment duration, and adherence hinders the attribution of dose–response mechanisms and further undermines study reproducibility. Third, heterogeneity and multiple biases are substantial. Pooled analyses mixing RCTs with retrospective cohorts result in high confounding; in particular, uncontrolled confounding by indication and immortal time bias in observational cohorts can easily inflate the survival benefit of Chinese herbal medicine at the algorithmic level. Fourth, high-value endpoint data are systematically missing. Core indicators for assessing net clinical benefit, such as toxicity, dose intensity, and quality of life, cannot be quantitatively synthesized due to inconsistent reporting standards. Finally, the presence of publication bias weakens the authenticity and generalizability of the evidence. Future research should adopt a multipronged approach across four dimensions. At the data and reporting level, individual participant data sharing and prospective data collection should be promoted to reduce measurement errors, and intervention details must be disclosed thoroughly in accordance with standardized guidelines to resolve the “black box” problem. At the methodological level, observational studies must use time-dependent methods such as landmark analysis and clone–censor–weight approaches to control bias, and RCTs and cohort studies should be analyzed separately to curb heterogeneity; concurrently, a core outcome set encompassing survival, toxicity, dose intensity, and quality of life should be established. In terms of study design, large-sample, multicenter, high-quality randomized trials are urgently needed to verify the true survival benefit of traditional Chinese medicine. Simultaneously, a dual prospective registration mechanism should be mandated within the review and publication system to curb selective reporting at its source, and the unbiased publication of negative results should be encouraged, thereby reshaping a reliable evidence chain for traditional Chinese medicine as an adjuvant therapy in oncology.

## 5 Conclusion

This study demonstrates that combining traditional Chinese medicine with chemotherapy is associated with improved OS and PFS in advanced non-small cell lung cancer, with benefits most evident during the active treatment phase. The findings support the concurrent integration of traditional Chinese medicine into standard chemotherapy regimens to enhance patient outcomes. Further research with standardized protocols is warranted to confirm these results and optimize treatment strategies.

## Data Availability

The raw data supporting the conclusions of this article will be made available by the authors, without undue reservation.
