# Supplementary material for: Long-term efficacy of traditional Chinese medicine combined with chemotherapy for advanced non-small cell lung cancer: a systematic review and meta-analysis of reconstructed individual patient data over 3 years
Source: Front Pharmacol. 2026 Jul 2;17:1818515. doi: 10.3389/fphar.2026.1818515 (PMC13373588; doi:10.3389/fphar.2026.1818515)
Supplement: Supplementary file 1 [file Supplementaryfile1.docx]

Supplementary Material

# Supplementary Figures and Tables

## Supplementary Figures


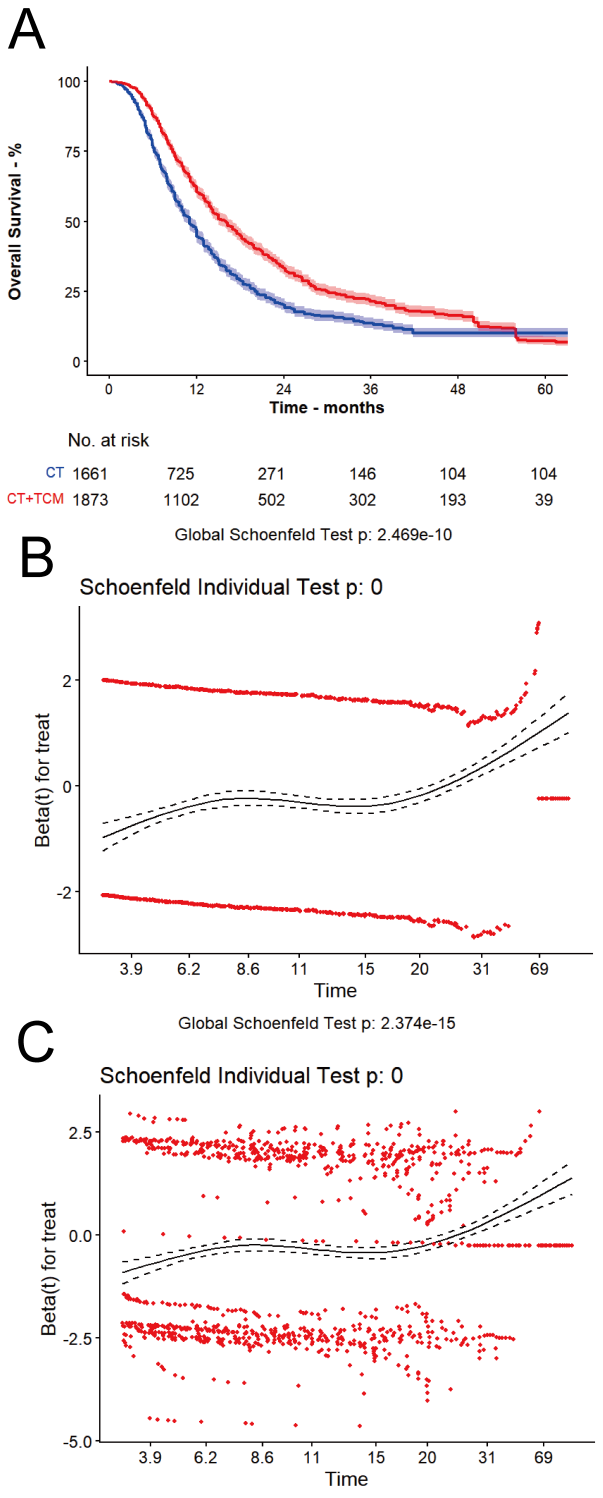


FigureS 1 Assessment of proportional hazards assumption for overall survival. (A) Reconstructed overall survival (OS) curves of all patients. (B) Schoenfeld residual plot for the treatment effect from the frailty Cox model. (C) Schoenfeld residual plot for the treatment effect from the stratified Cox model.


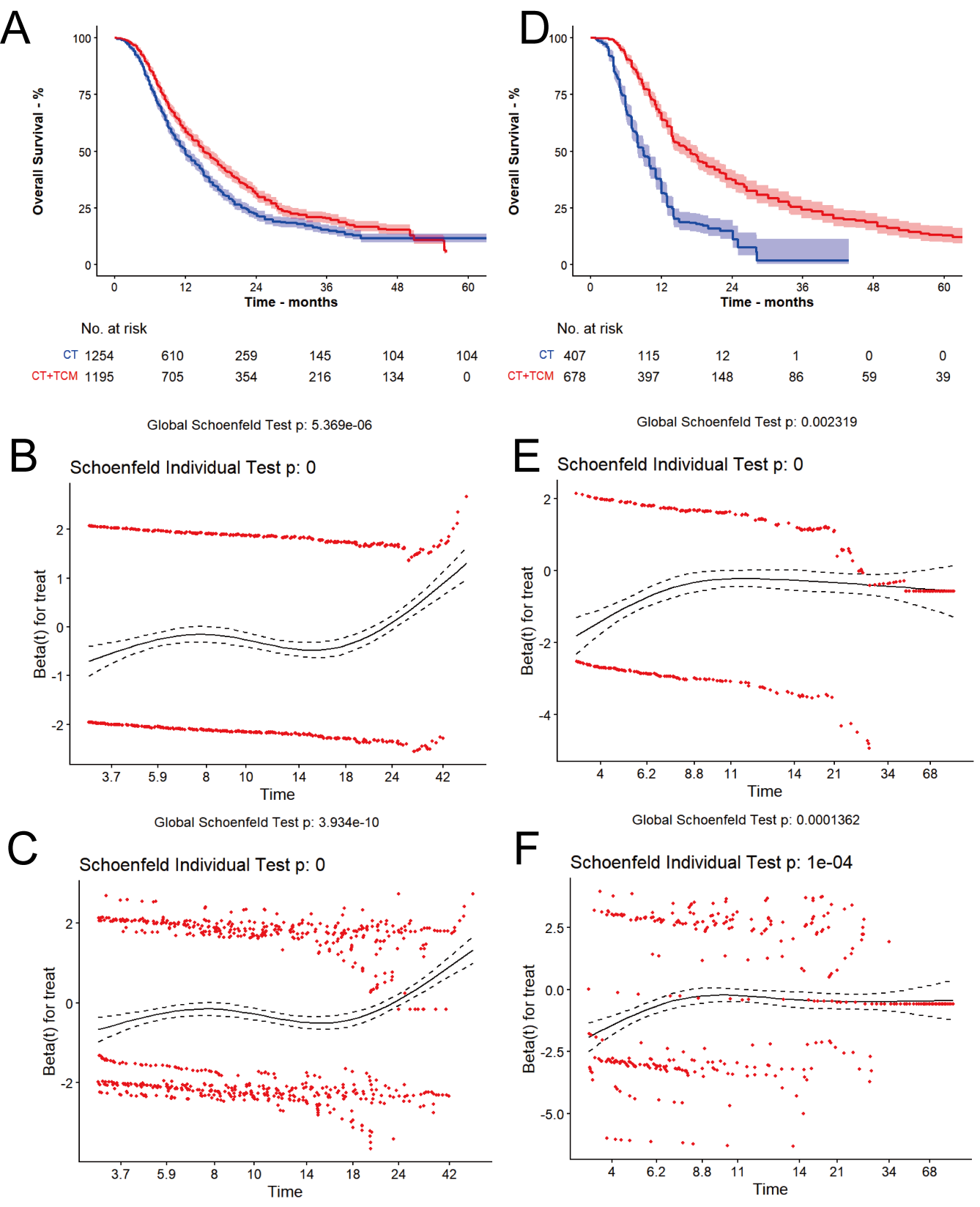


FigureS 2 (A) Reconstructed OS curve of fixed-regimen treatment group. (B) Schoenfeld residual plot of the fixed-regimen treatment group from the frailty Cox model. (C) Schoenfeld residual plot of the fixed-regimen treatment group from the stratified Cox model. (D) Reconstructed OS curve of personalized treatment group. (E) Schoenfeld residual plot of the personalized treatment group from the frailty Cox model. (F) Schoenfeld residual plot of the personalized treatment group from the stratified Cox model.


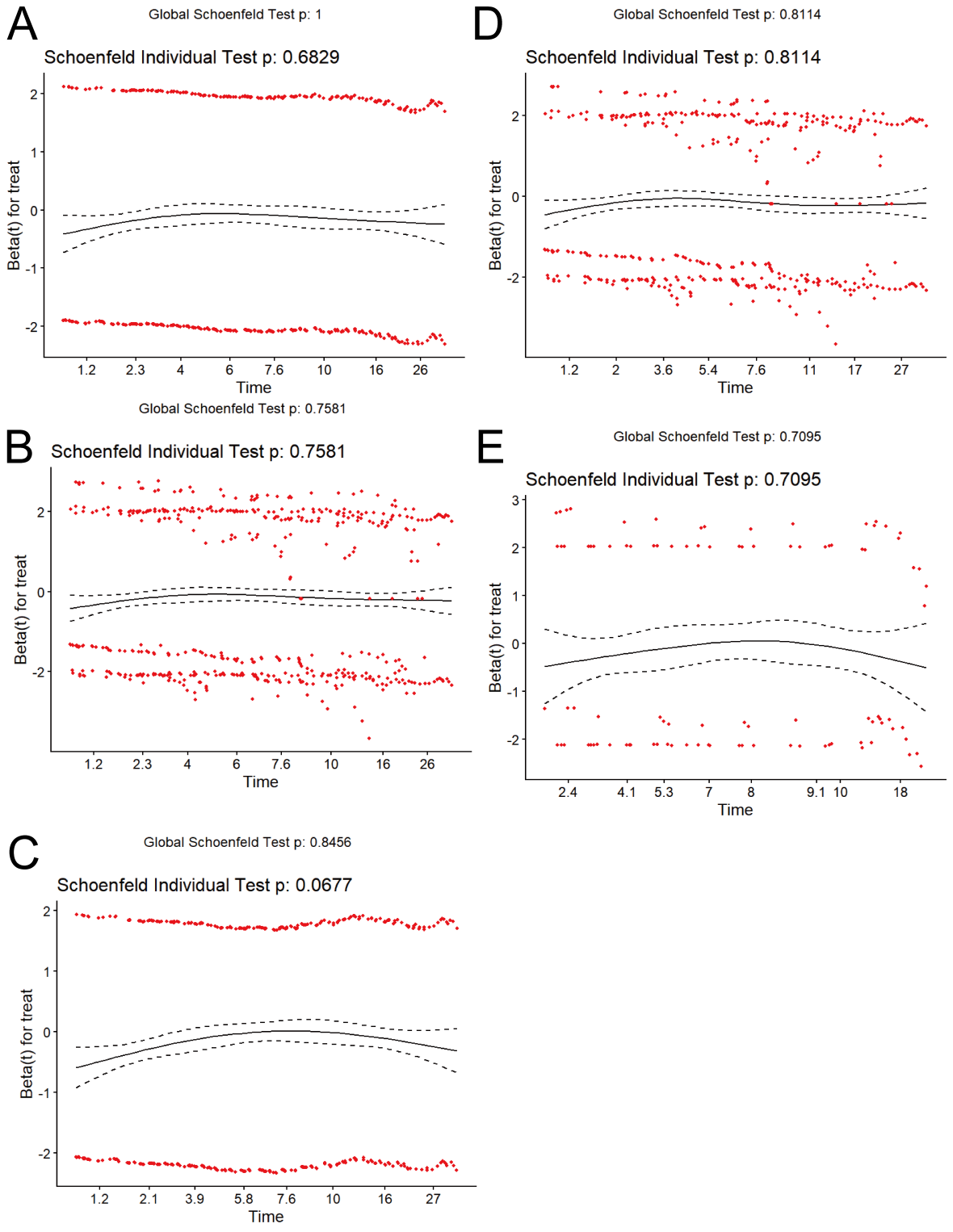


FigureS 3 Assessment of proportional hazards assumption for progression-free survival(PFS). (A) Schoenfeld residual plot for the treatment effect from the frailty Cox model. (B) Schoenfeld residual plot for the treatment effect from the stratified Cox model. (C) Schoenfeld residual plot of the fixed-regimen treatment group from the frailty Cox model. (D) Schoenfeld residual plot of the fixed-regimen treatment group from the stratified Cox model. (E) Schoenfeld residual plot of the personalized treatment group from the stratified Cox. Schoenfeld residual plot not shown for this subgroup because the small number of studies precluded its generation in the frailty Cox model.


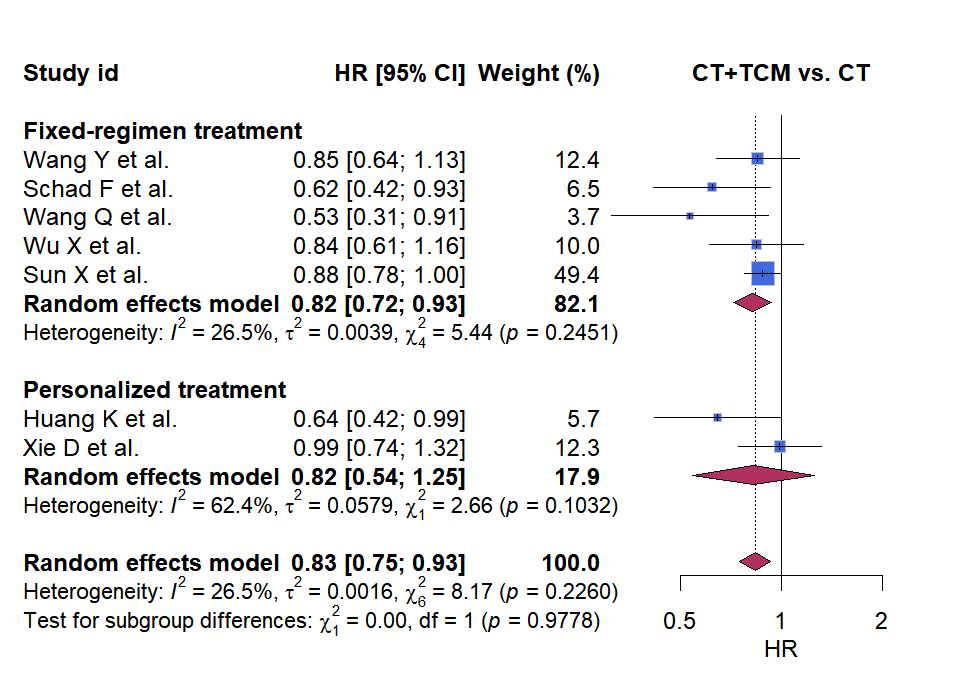


FigureS 4 Two-stage sensitivity meta-analysis forest plot for PFS (CT+TCM vs. CT), stratified by fixed-regimen and personalized treatment subgroups. The overall random-effects model showed a significant benefit for CT+TCM with low heterogeneity (HR = 0.83, 95% CI 0.75–0.93, I² = 26.5%). Within subgroups, the pooled effect was (HR = 0.82, 95% CI 0.72–0.93, I² = 26.5%) for the fixed-regimen group and (HR = 0.82, 95% CI 0.54–1.25, I² = 62.4%) for the personalized treatment group. The test for subgroup differences was not significant (p = 0.9778).


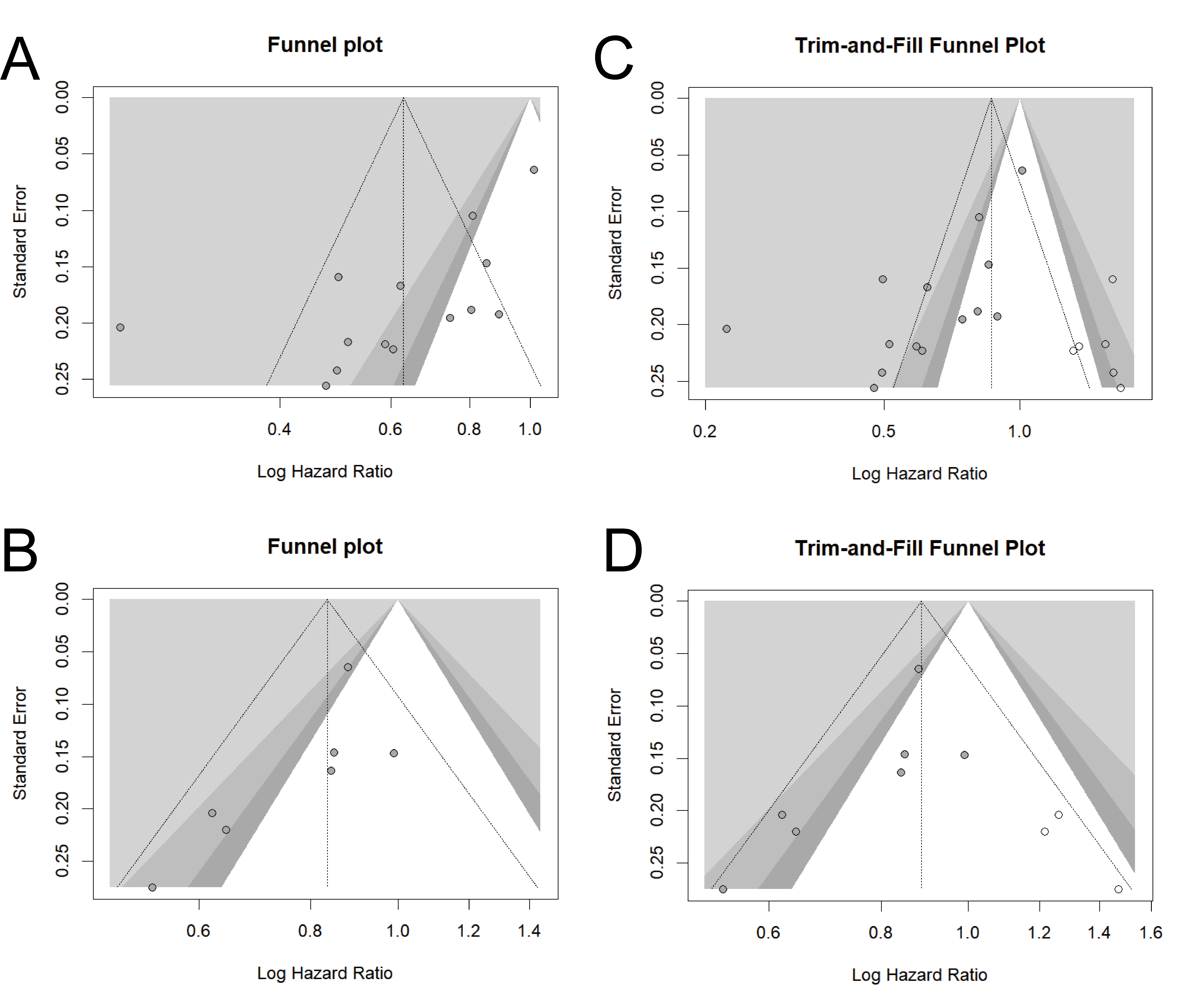


FigureS 5 Funnel plots for publication bias assessment. (A) Funnel plot of included studies for OS. Egger’s test for OS confirmed small-study effects (t = −3.83, df = 12, p = 0.0024) (B) Funnel plot of included studies for PFS. formal testing for PFS was not feasible due to insufficient studies (n = 7). (C) Trim-and-fill adjustment, the pooled HRs for OS (HR = 0.86, 95% CI 0.67–1.12, p = 0.26, I² = 88.9%). (D) Trim-and-fill adjustment, the pooled HRs for PFS (HR = 0.89, 95% CI 0.77–1.03, p = 0.11, I² = 49.4%). Each circle represents an individual study; the vertical dashed line indicates the pooled effect estimate, and the diagonal lines denote the pseudo 95% confidence limits.

## Supplementary Tables

| Author | Year | OS | PFS |
| --- | --- | --- | --- |
| Zhou D et al. | 2005 | 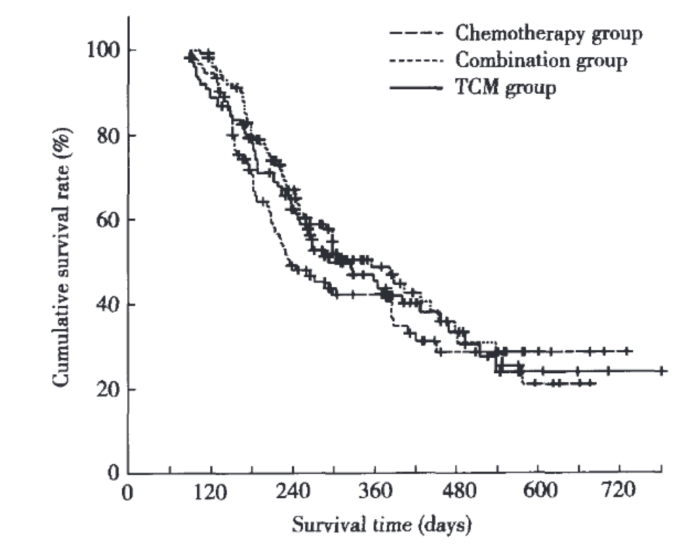 |  |
| Chen Y et al. | 2008 | 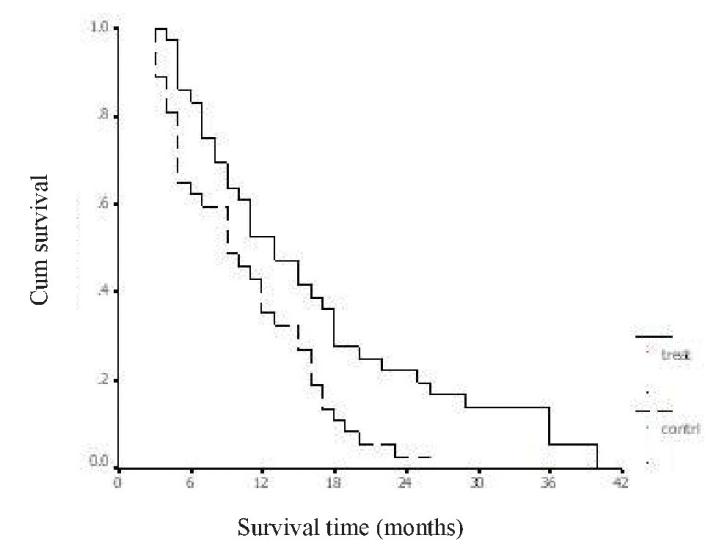 |  |
| Guo H et al. | 2011 | 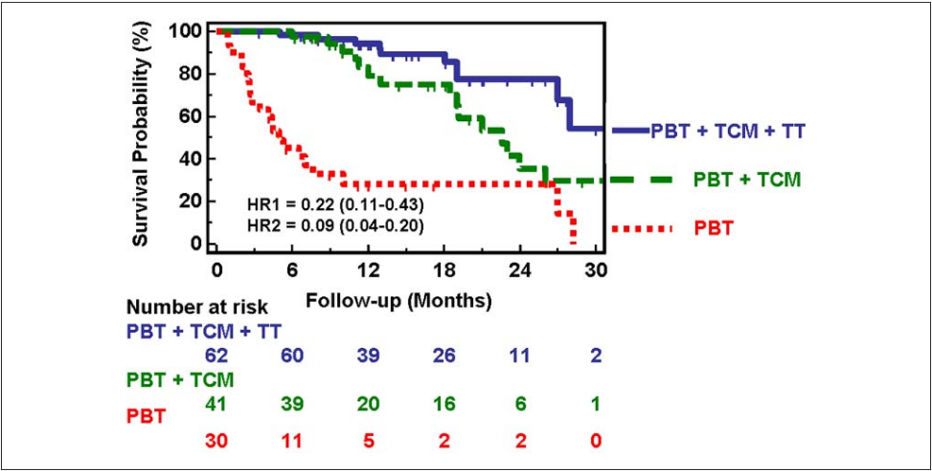 |  |
| Xu Z et al. | 2011 | 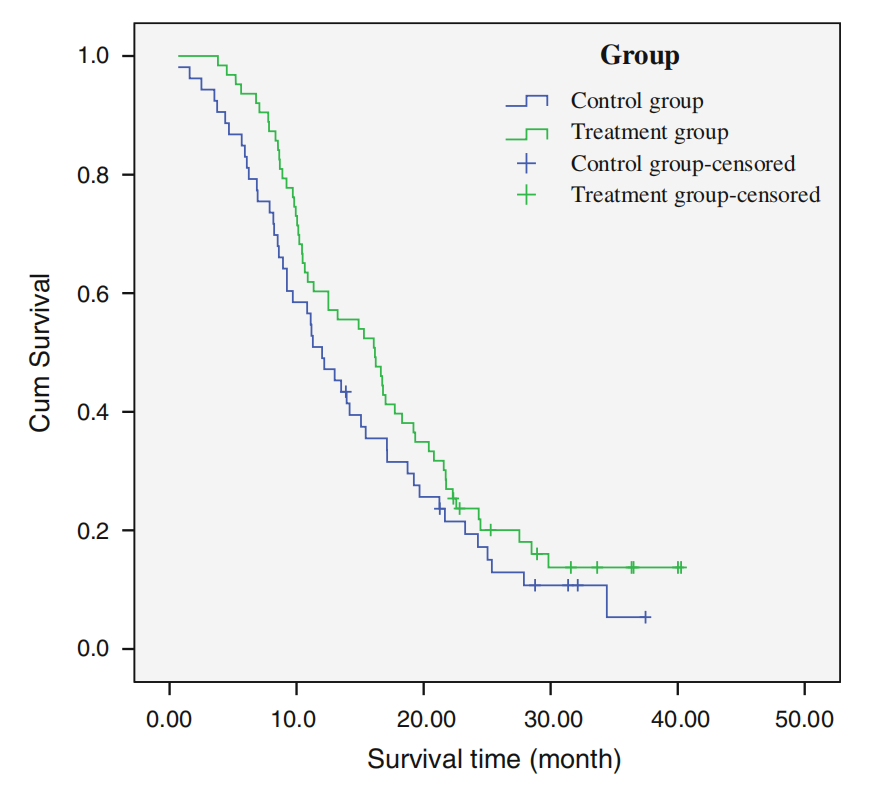 |  |
| Guo L et al. | 2012 | 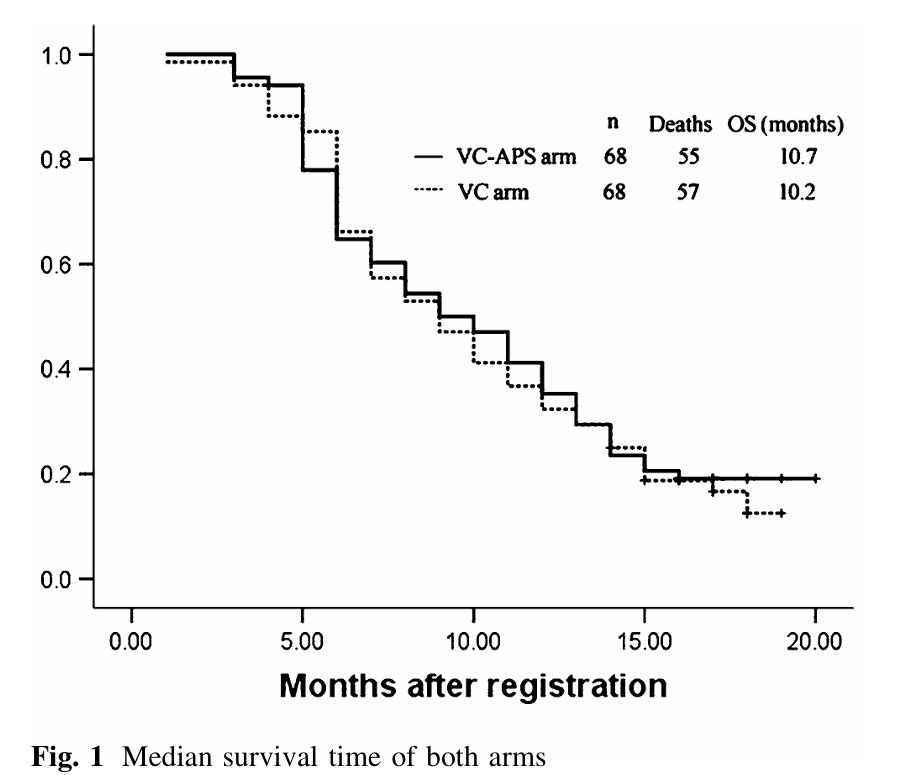 |  |
| Xie D et al. | 2012 | 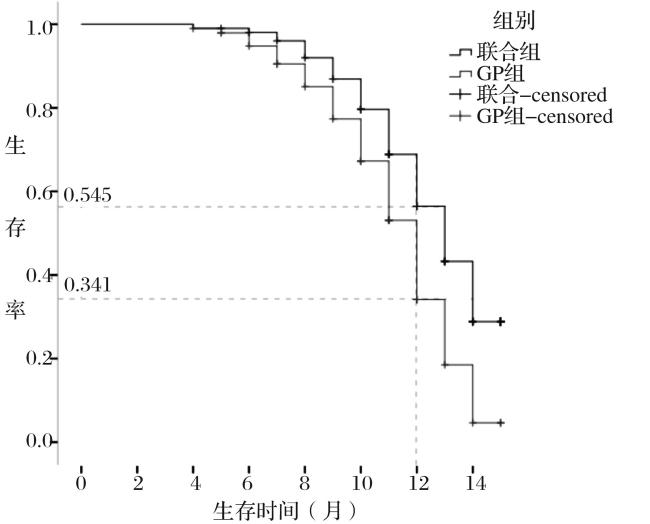 | 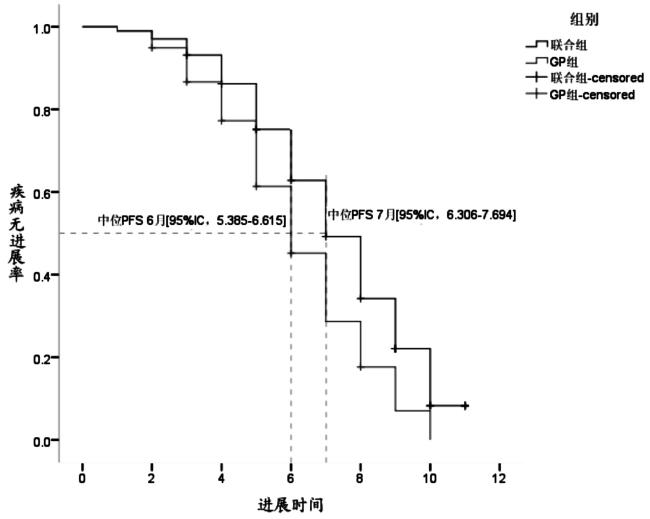 |
| Wang Z et al. | 2013 | 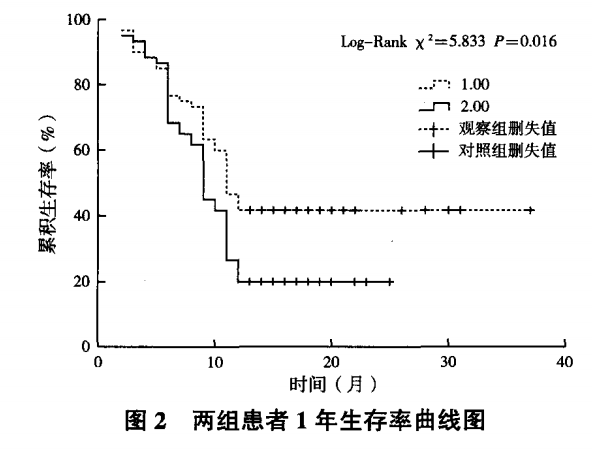 | 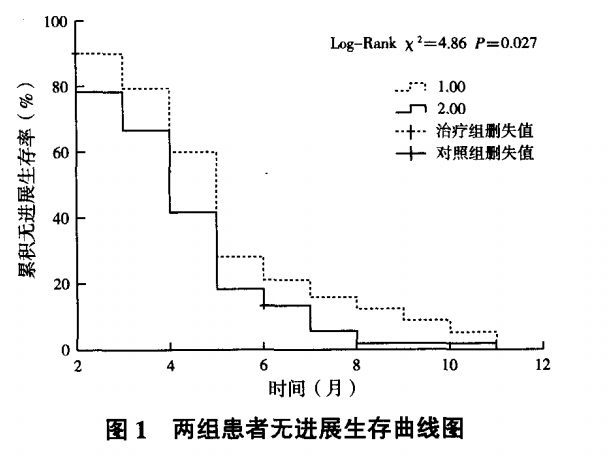 |
| Rong Z et al. | 2014 | 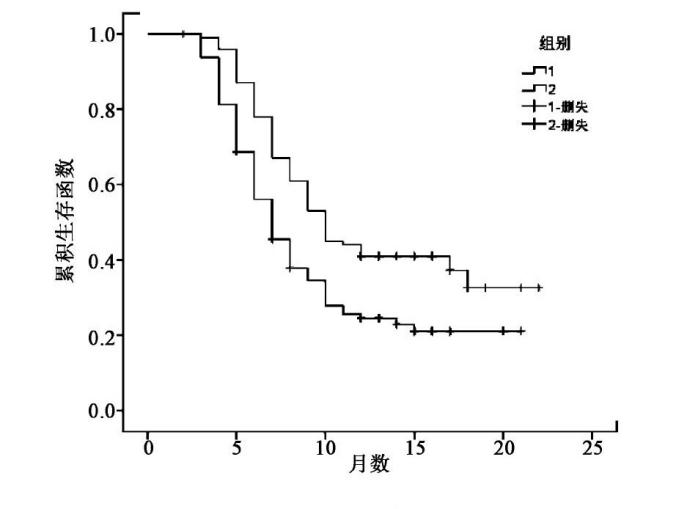 |  |
| Li K et al. | 2016 | 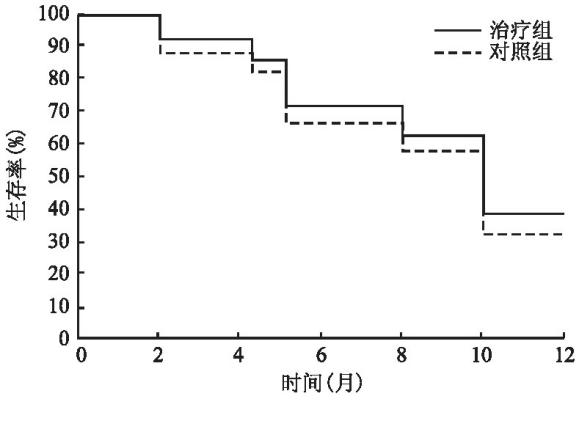 |  |
| Wang Y et al. | 2016 | 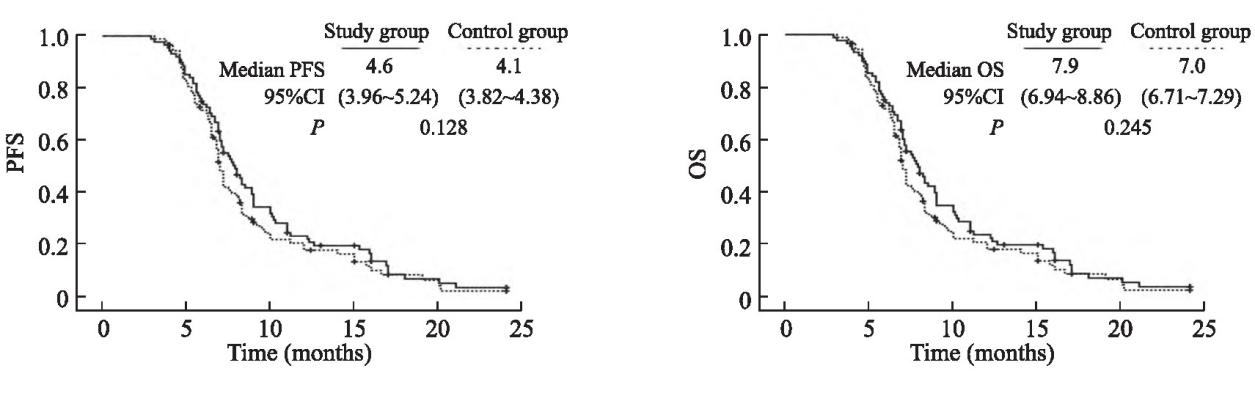 | |
| Schad F et al. | 2018 | 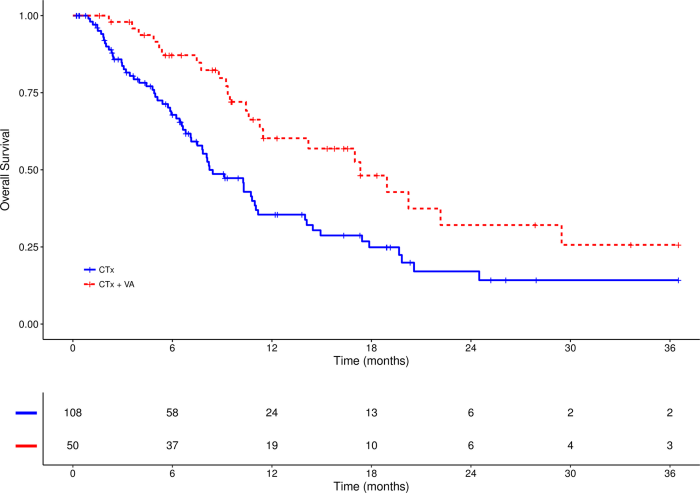 | 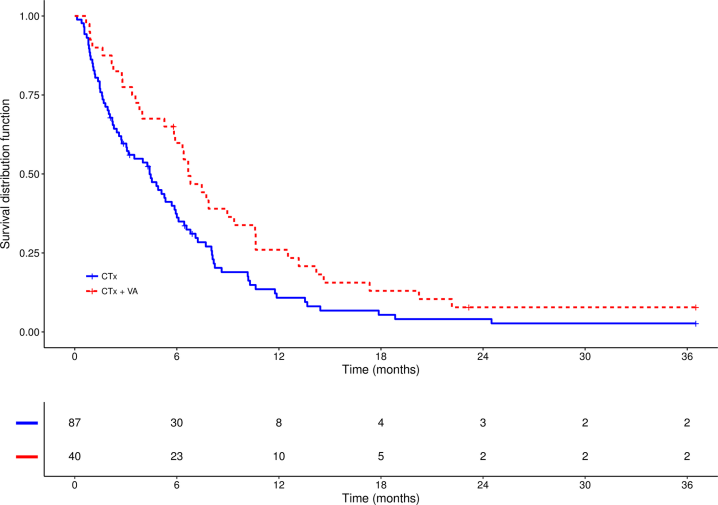 |
| Zhang Y et al. | 2018 | 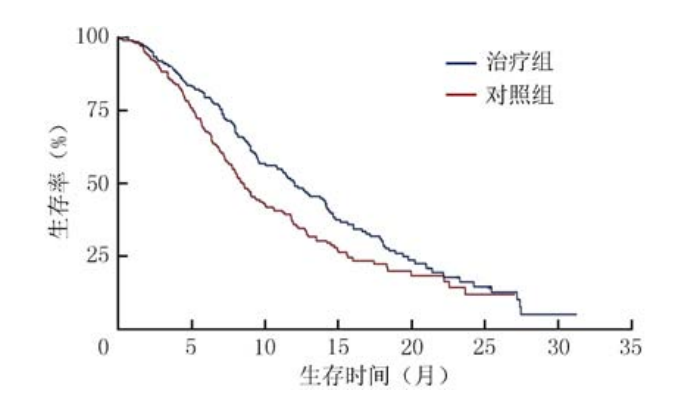 |  |
| Wu X et al. | 2018 |  | 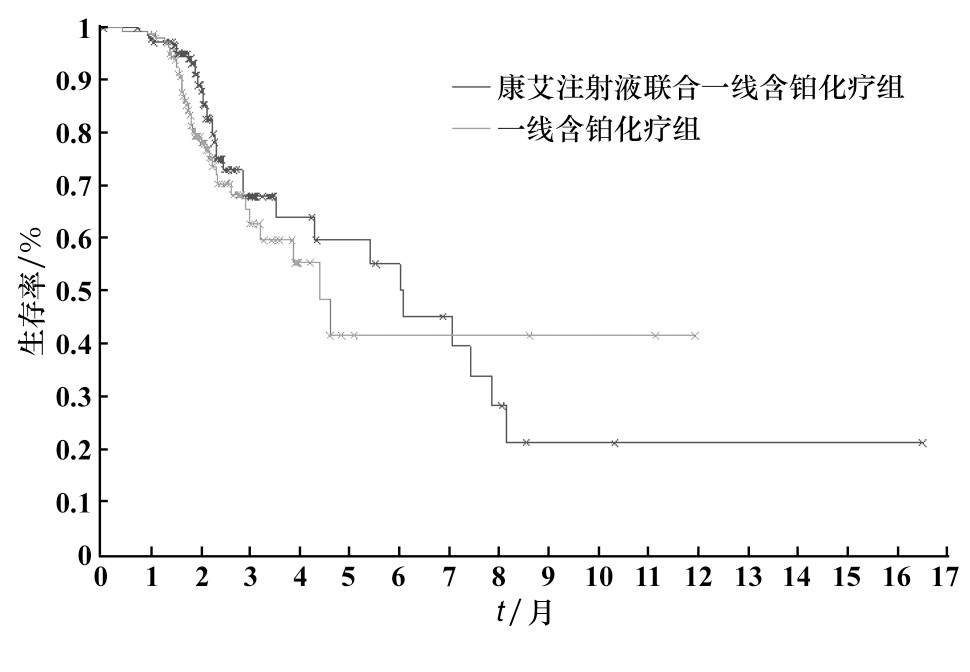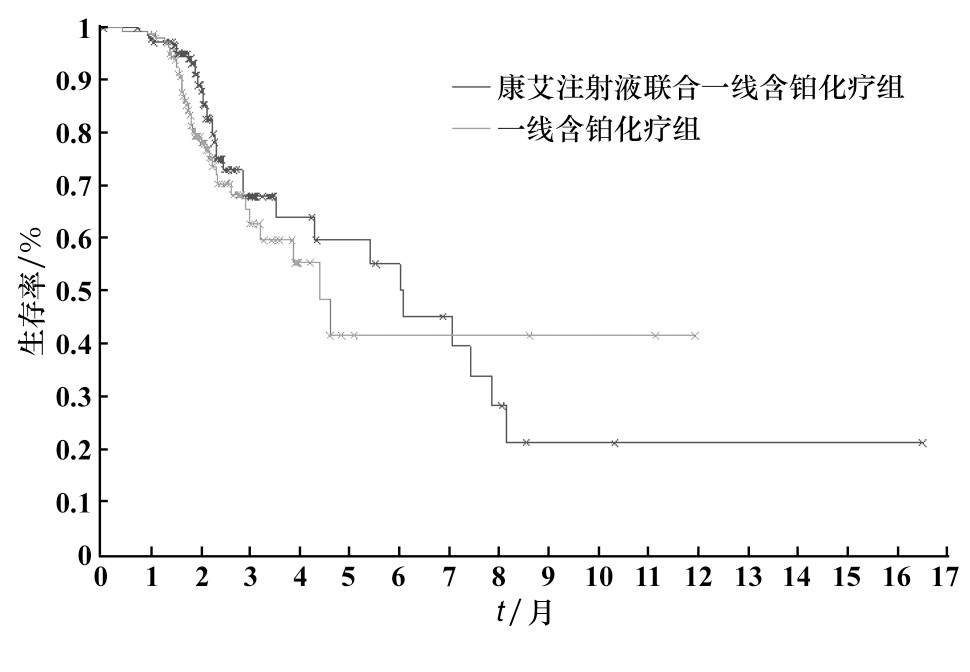 |
| Wang Q et al. | 2018 |  | 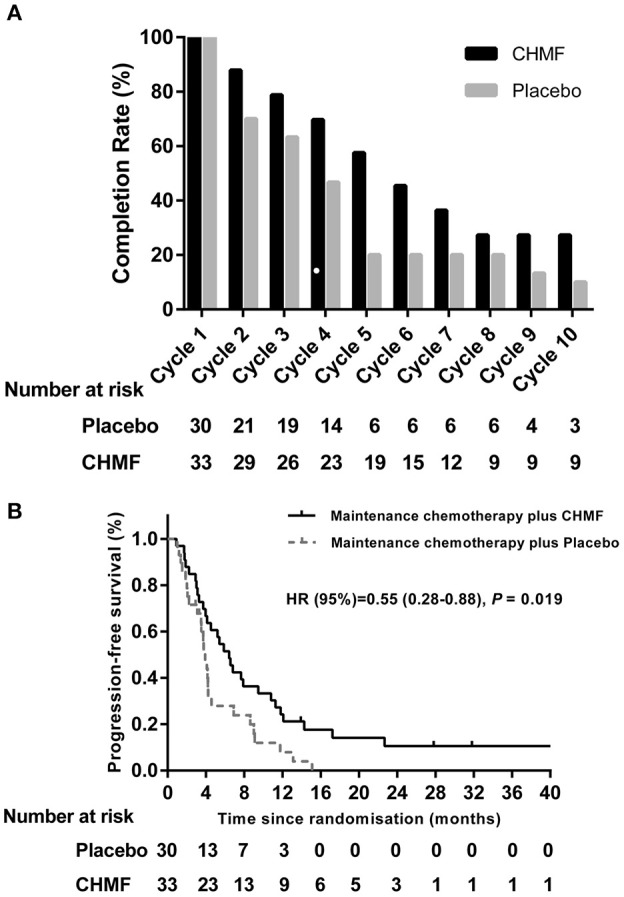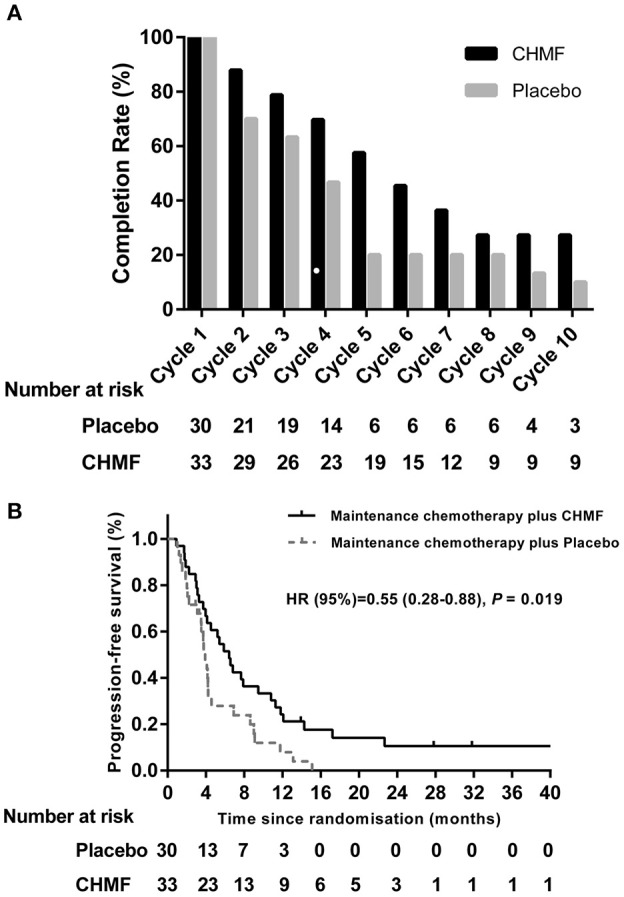 |
| Huang K et al. | 2021 | 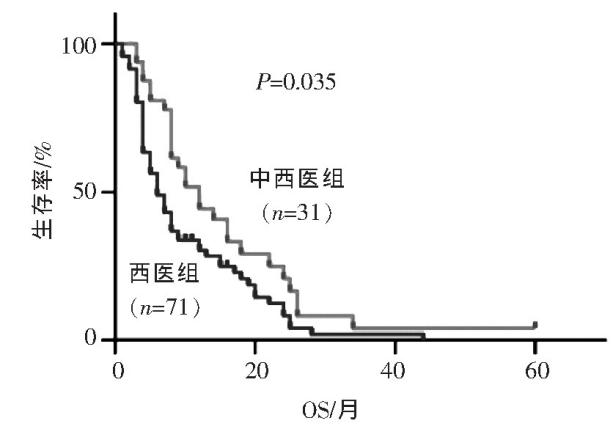 | 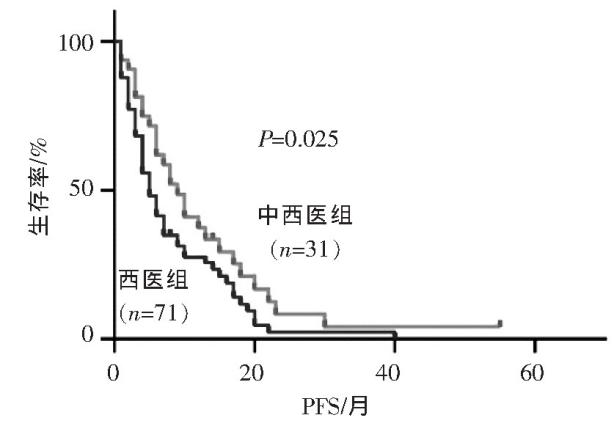 |
| Sun X et al. | 2022 | 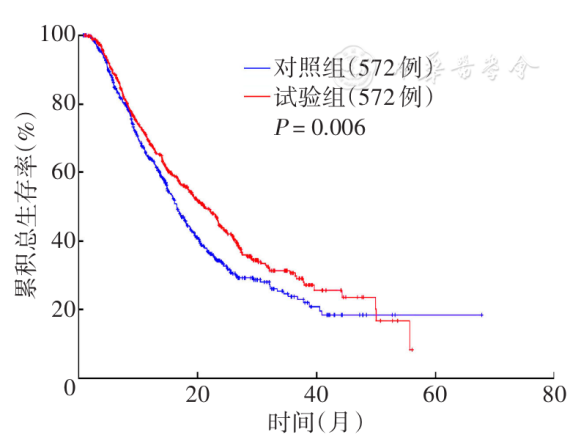 | 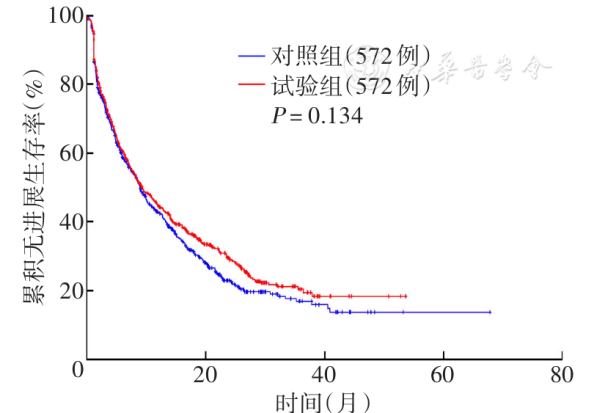 |
| Guo H et al. | 2023 | 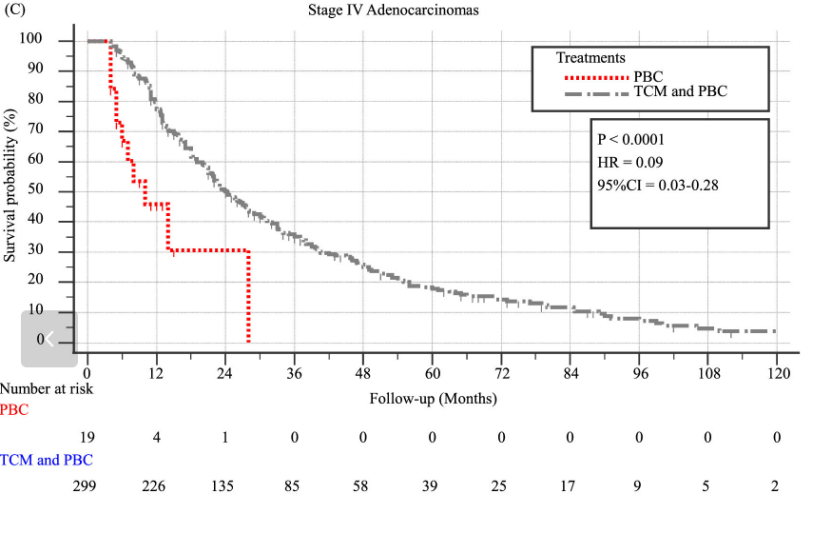 |  |
| Chang F et al. | 2024 | 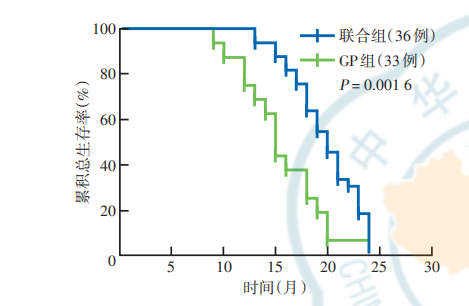 |  |
